# Python/NEURON code for simulating biophysically realistic thalamocortical dynamics during sleep

**DOI:** 10.1016/j.simpa.2024.100667

**Published:** 2024-06-03

**Authors:** Christian G. Fink, Pavel Sanda, Logan Bayer, Eroma Abeysinghe, Maxim Bazhenov, Giri P. Krishnan

**Affiliations:** a Gonzaga University, Spokane, WA, USA; b Institute of Computer Science of the Czech Academy of Sciences, Prague, Czech Republic; c Georgetown University, Washington, DC, USA; d Georgia Institute of Technology, Atlanta, GA, USA; e University of California, San Diego, CA, USA

**Keywords:** Computational neuroscience, Sleep, Neuromodulation, NEURON

## Abstract

Understanding the function of sleep and its associated neural rhythms is an important goal in neuroscience. While many theoretical models of neural dynamics during sleep exist, few include the effects of neuromodulators on sleep oscillations and describe transitions between sleep and wake states or different sleep stages. Here, we started with a C++-based thalamocortical network model that describes characteristic thalamic and cortical oscillations specific to sleep. This model, which includes a biophysically realistic description of intrinsic and synaptic channels, allows for testing the effects of different neuromodulators, intrinsic cell properties, and synaptic connectivity on neural dynamics during sleep. We present a complete reimplementation of this previously-published sleep model in the standardized NEURON/Python framework, making it more accessible to the wider scientific community.

**Table:** 

Code metadata	
Current code version	v1
Permanent link to code/repository used for this code version	https://github.com/SoftwareImpacts/SIMPAC-2024-84
Permanent link to Reproducible Capsule	
Legal Code License	GPL-2
Code versioning system used	None
Software code languages, tools, and services used	Python, NEURON, MPI
Compilation requirements, operating environments & dependencies	NumPy, SciPy, and Matplotlib packages for Python
If available Link to developer documentation/manual	See readme.md at ModelDB link
Support email for questions	finkt@gonzaga.edu

## 1. Introduction

The phenomenon of sleep is mysterious: nearly all animals exhibit some form of sleep, yet we cannot say exactly why this must be so. Many clues have surfaced over the past few decades, with neural activity during mammalian sleep exhibiting a rich repertoire of rhythms associated with memory consolidation [1,2], toxin clearance [3], and regulation of synaptic strength [4]. Different stages of sleep feature characteristic neural activity patterns, with sleep spindles prominent during non-REM Stage 2 (associated with memory consolidation [5]), slow waves dominating non-REM Stage 3 (associated with memory consolidation [6] and clearance of metabolic waste [7]), and wake-like alpha activity characteristic of REM sleep. Such neural activity is heavily influenced by the concentration of various neuromodulators (such as acetylcholine, histamine, norepinephrine, etc.) that modulate the activity of neurons at the cellular and network levels.

Disentangling the effects of various neuromodulators on neural activity, and how this influences sleep activity and transitions between sleep stages, is an active area of exploration in neuroscience. Experimental investigation of these questions is ongoing [8-10], but it is often difficult or impossible to determine levels of neuromodulators across the brain with current technology. Computationally modeling the effects of neuromodulators on neural activity during sleep can provide useful models to guide experimental investigation. One such computational model that examines the effect of neuromodulation on sleep activity was proposed by Krishnan et al. [11]. It simulates the effects of varying concentrations in acetylcholine, histamine, and GABA on neural activity during wake, N2, N3, and REM sleep. The model was based on extensive previous modeling and experimental works [12-24], and several subsequent studies have utilized this model’s approach to simulate neuromodulation in their work [25-30]. It is also one of the few biophysically realistic, detailed individual neuron-based (as opposed to mean field or firing rate-based) models of neural dynamics during sleep. We have re-implemented it in a more accessible framework that allows other researchers to easily modify the model to explore their own lines of enquiry.

## 2. Software

**Fig. 1.**
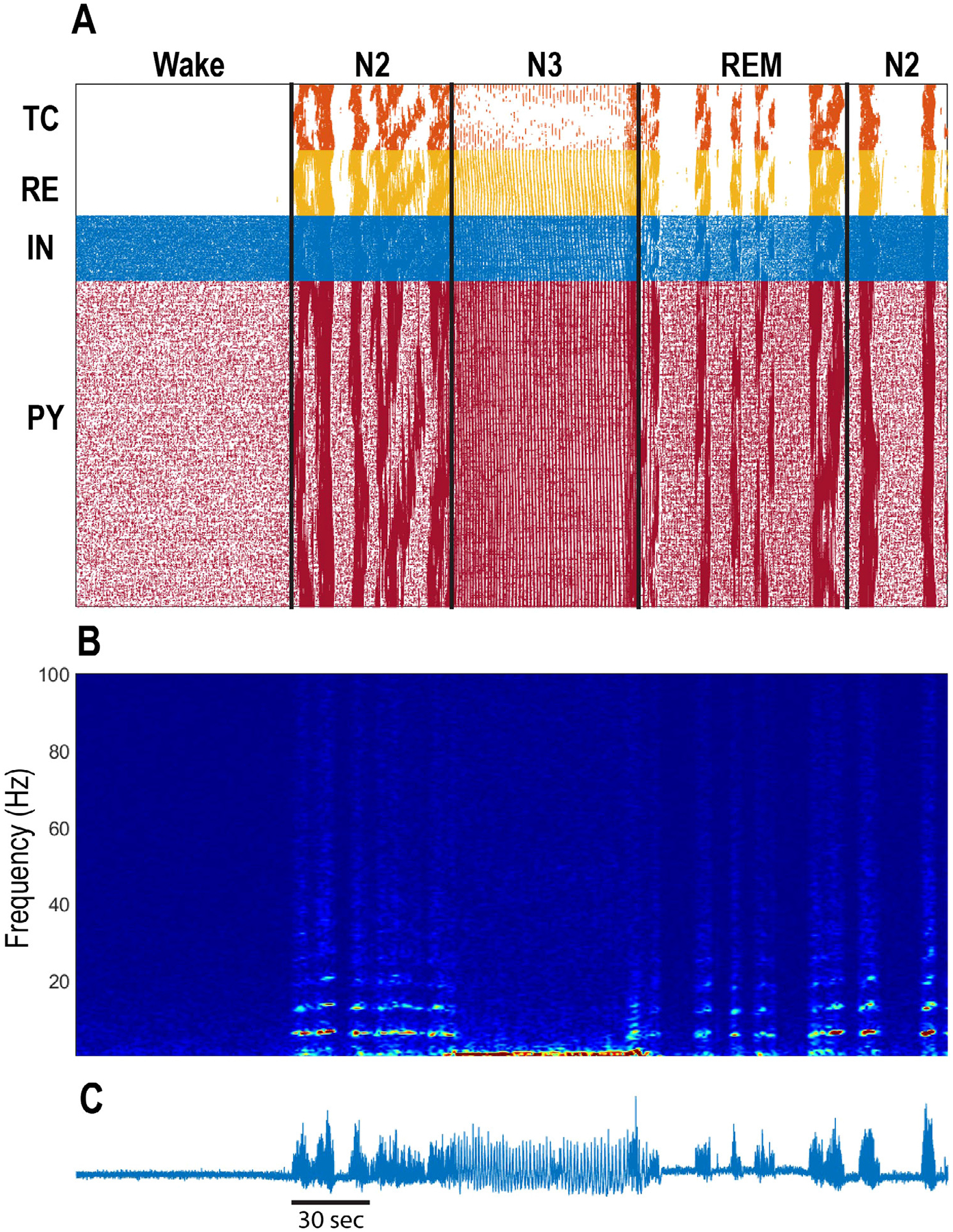
Simulated sleep cycles In a thalamocortical model. (A) Raster plot of network activity for wake, N2, N3, REM, and N2 sleep stages. Different sleep stages were generated my modulating acetylcholine, histamine, and GABA. (B) Spectrogram of simulated cortical local field potential. Note the spindle rhythms in N2 and the slow waves in N3. (C) Simulated cortical local field potential, computed by summing intracellular voltage traces.

The model involves a simulated thalamocortical loop, with 500 cortical pyramidal (PY) neurons, 100 cortical inhibitory (INH) neurons, 100 thalamocortical (TC) relay neurons, and 100 thalamic reticular nucleus (RE) neurons. Krishnan et al. [11] used this model to show how plausible changes in neuromodulation could give rise to the neural rhythms characteristic of different stages of sleep, and they used their results to suggest that differences in neural rhythms between human and animal recordings were primarily due to differences in acetylcholine concentration. The model’s code was originally written in C++, which resulted in fast simulation times, but it is hardly accessible for newcomers and results in slow development of new features. To ease this situation we present here the model from [11], ported to the widely used Python-based NEURON simulation environment [31]. Fig. 1 depicts the main simulation results from [11], but regenerated using NEURON. NEURON is one of the leading simulation environments in the field of computational neuroscience, allowing users to develop models at the level of biophysical detail using Python, without having to worry about numerical implementation. NEURON auto-generates C code to take care of such numerical details, so that simulations are fast without requiring inordinate development time. NEURON also includes libraries for parallel computation, and our code is fully parallelized [32]. The 360,000 ms simulation shown in Fig. 1 took approximately 15 min to run with 128 cores on the Neuroscience Gateway Computing (NSG) cluster [33]. Commands for submitting this simulation to NSG are included in the repository. Further, the model is also available at the Cybershuttle site (https://neuroscience.cybershuttle.org) [34], which allows for tracking simulations and parameter sweeps.

The code is organized around four Python files: config.py defines all simulation parameters, cell_classes.py defines classes for the four kinds of neurons (PY, INH, TC, and RE), network_class.py defines a class prescribing network connectivity, and main.py initializes and runs the simulation. The most important ancillary files are the so-called “mod files,” which consist of high-level NMODL code specifying various mathematical details of the model (such as equations describing the biophysics of various kinds of synapses). mod files are translated into C code by NEURON before running a simulation (using the command nrnivmodl), which is how NEURON simulations can run so efficiently without the need to explicitly develop code in a low-level language like C or C++. In addition, two legacy files are included (interpxyz.hoc and calcrxc_a.hoc) for simulations in which the cortical LFP is calculated from biophysical first principles. These compute the spatial locations of all neuronal compartments and the simulated recording electrode, as well as the transfer resistance between the recording electrode and each compartment. The detailed instructions for installation and running are described in the README.md file of the software package.

## 3. Impact

Our NEURON simulation code serves as a foundation for other researchers interested in developing computational models of thalamocortical activity during wake and sleep. Additional capabilities such as simulating stochastically varying neuromodulator concentrations, memory consolidation, spindle-slow wave coupling, etc., are all much more readily developed in NEURON than in lower-level languages such as C++. One feature that we have added as a new feature in this software package is the calculation of local field potential (LFP) from biophysical first principles, as opposed to simply summing intracellular voltages. While summing intracellular voltages is a useful first approximation to the biophysical LFP, this method does not reflect the true source of extracellular potentials. Our NEURON implementation uses the method outlined in [35] to determine the contributions of all transmembrane currents to the net potential at the recording electrode. Given the widespread interest in how different sleep rhythms emerge and interact in different stages of sleep [36-38], the more realistic simulation of these rhythms offered by our model may be of significant interest to the broader scientific community. Other researchers may also be interested in using our “mod” files in their own NEURON models. For example, the mod file describing synaptic interactions between pyramidal cells (AMPA_D2.mod) is essential to the generation of cortical slow waves in our model, and it could be used in other cortical models for the same purpose.

More broadly, previous work has suggested that simulated sleep can improve deep learning paradigms. One flaw in modern artificial neural network performance is the phenomenon of “catastrophic forgetting,” in which the learning of new memories invariably degrades previously-learned memories. The human brain does not suffer from this problem to nearly the same extent, and it is thought that sleep is a major reason why. This has inspired efforts to simulate sleep in artificial neural networks, in order to mitigate catastrophic forgetting [29,39]. Our Python/NEURON implementation of this model of sleep will enable more nimble development of software to address the problem of catastrophic forgetting.

In the future, we plan to develop this model to simulate the effects of various neuromodulators on sleep rhythms [40-43]. Future development could include upgrading the code to be compatible with coreNEURON [44], which would allow it to run on GPU architectures.

## 4. Limitations

From a neuroscience point of view, this thalamocortical model is limited by the fact that it includes the effects of only a select group of neuromodulators (omitting norepinephrine, orexin, and serotonin, among others). However, incorporating these neuromodulators into the model would be relatively straightforward using NEURON. This network model also uses relatively simple one- or two-compartment models for individual neurons. The original C++ code further simplified the two-compartment cortical cells by omitting capacitance from the axosomatic compartment, which helped to speed the simulation numerically [12,45]. In porting the model to NEURON, we included capacitance in the axosomatic compartment, which required that associated conductances be downscaled by a factor of 3/4 in order to give similar qualitative results to the C++ code. The NEURON code is therefore not an exact translation of the C++ code, but comparing Fig. 1 of this paper to Fig. 2 in [11] demonstrates the overall fidelity of the NEURON code to the original.

## 5. Conclusion

The thalamocortical model by Krishnan et al. [11] is one of the few biophysically realistic network models of different sleep stages in the computational neuroscience literature. Here we have re-written the code using the Python-based NEURON framework, enabling other researchers to efficiently adapt the model to their own research questions. Anyone interested in computational modeling of sleep rhythms, in particular their dependence on neuromodulatory tone and their possible effects on memory consolidation, will find this software useful.

## References

[1] Girardeau G, Lopes-Dos-Santos V, Brain neural patterns and the memory function of sleep, Science 374 (6567) (2021) 560–564. 34709916 10.1126/science.abi8370 PMC7611961

[2] Fernandez LM, Lüthi A, Sleep spindles: mechanisms and functions, Physiol. Rev 100 (2) (2020) 805–868. 31804897 10.1152/physrev.00042.2018

[3] Fultz NE, Bonmassar G, Setsompop K, Stickgold RA, Rosen BR, Polimeni JR, Lewis LD, Coupled electrophysiological, hemodynamic, and cerebrospinal fluid oscillations in human sleep, Science 366 (6465) (2019) 628–631. 31672896 10.1126/science.aax5440 PMC7309589

[4] Timofeev I, Chauvette S, Sleep slow oscillation and plasticity, Curr. Opin. Neurobiol 44 (2017) 116–126. 28453998 10.1016/j.conb.2017.03.019

[5] Ngo H-V, Fell J, Staresina B, Sleep spindles mediate hippocampal-neocortical coupling during long-duration ripples, ELife 9 (2020) e57011. 32657268 10.7554/eLife.57011 PMC7363445

[6] Born J, Slow-wave sleep and the consolidation of long-term memory, World J. Biol. Psychiatry 11 (sup1) (2010) 16–21. 20509828 10.3109/15622971003637637

[7] Picchioni D, Özbay PS, Mandelkow H, de Zwart JA, Wang Y, van Gelderen P, Duyn JH, Autonomic arousals contribute to brain fluid pulsations during sleep, Neuroimage 249 (2022) 118888. 35017126 10.1016/j.neuroimage.2022.118888 PMC11395500

[8] McCormick DA, Neurotransmitter actions in the thalamus and cerebral cortex and their role in neuromodulation of thalamocortical activity, Prog. Neurobiol 39 (4) (1992) 337–388. 1354387 10.1016/0301-0082(92)90012-4

[9] Samanta A, Alonso A, Genzel L, Memory reactivations and consolidation: considering neuromodulators across wake and sleep, Curr. Opin. Physiol 15 (2020) 120–127.

[10] Falup-Pecurariu C, Diaconu S, Tint D, Falup-Pecurariu O, Neurobiology of sleep, Exp. Therapeutic Med 21 (3) (2021) 1–1. 10.3892/etm.2021.9703 PMC7851648 33603879

[11] Krishnan GP, Chauvette S, Shamie I, Soltani S, Timofeev I, Cash SS, Halgren E, Bazhenov M, Cellular and neurochemical basis of sleep stages in the thalamocortical network, ELife 5 (2016) e18607. 27849520 10.7554/eLife.18607 PMC5111887

[12] Bazhenov M, Timofeev I, Steriade M, Sejnowski TJ, Model of thalamocortical slow-wave sleep oscillations and transitions to activated states, J. Neurosci 22 (19) (2002) 8691–8704. 12351744 10.1523/JNEUROSCI.22-19-08691.2002 PMC6757797

[13] Fuentealba P, Timofeev I, Bazhenov M, Sejnowski TJ, Steriade M, Membrane bistability in thalamic reticular neurons during spindle oscillations, J. Neurophysiol 93 (1) (2005) 294–304. 15331618 10.1152/jn.00552.2004 PMC2915789

[14] Bazhenov M, Timofeev I, Intrinsic and synaptic mechanisms of cortical active states generation during slow-wave sleep, in: Mechanisms of Spontaneous Active States in the Neocortex, 2007, pp. 1–22.

[15] Bonjean M, Baker T, Lemieux M, Timofeev I, Sejnowski T, Bazhenov M, Corticothalamic feedback controls sleep spindle duration in vivo, J. Neurosci 31 (25) (2011) 9124–9134. 21697364 10.1523/JNEUROSCI.0077-11.2011 PMC3131502

[16] Bazhenov M, Lonjers P, Skorheim S, Bedard C, Destexhe A, Non-homogeneous extracellular resistivity affects the current-source density profiles of up-down state oscillations, Phil. Trans. R. Soc. A 369 (1952) (2011) 3802–3819. 21893529 10.1098/rsta.2011.0119 PMC3263778

[17] Bonjean M, Baker T, Bazhenov M, Cash S, Halgren E, Sejnowski T, Interactions between core and matrix thalamocortical projections in human sleep spindle synchronization, J. Neurosci 32 (15) (2012) 5250–5263. 22496571 10.1523/JNEUROSCI.6141-11.2012 PMC3342310

[18] Chen J-Y, Chauvette S, Skorheim S, Timofeev I, Bazhenov M, Interneuron-mediated inhibition synchronizes neuronal activity during slow oscillation, J. Physiol 590 (16) (2012) 3987–4010. 22641778 10.1113/jphysiol.2012.227462 PMC3476644

[19] Lemieux M, Chen J-Y, Lonjers P, Bazhenov M, Timofeev I, The impact of cortical deafferentation on the neocortical slow oscillation, J. Neurosci 34 (16) (2014) 5689–5703. 24741059 10.1523/JNEUROSCI.1156-13.2014 PMC3988418

[20] Chen J-Y, Lonjers P, Lee C, Chistiakova M, Volgushev M, Bazhenov M, Heterosynaptic plasticity prevents runaway synaptic dynamics, J. Neurosci 33 (40) (2013) 15915–15929. 24089497 10.1523/JNEUROSCI.5088-12.2013 PMC3787503

[21] Mak-McCully RA, Deiss SR, Rosen BQ, Jung K-Y, Sejnowski TJ, Bastuji H, Rey M, Cash SS, Bazhenov M, Halgren E, Synchronization of isolated downstates (K-complexes) may be caused by cortically-induced disruption of thalamic spindling, PLoS Comput. Biol 10 (9) (2014) e1003855. 25255217 10.1371/journal.pcbi.1003855 PMC4177663

[22] Volgushev M, Chen J-Y, Ilin V, Goz R, Chistiakova M, Bazhenov M, Partial breakdown of input specificity of STDP at individual synapses promotes new learning, J. Neurosci 36 (34) (2016) 8842–8855. 27559167 10.1523/JNEUROSCI.0552-16.2016 PMC4995300

[23] Bannon NM, Chistiakova M, Chen J-Y, Bazhenov M, Volgushev M, Adenosine shifts plasticity regimes between associative and homeostatic by modulating heterosynaptic changes, J. Neurosci 37 (6) (2017) 1439–1452. 28028196 10.1523/JNEUROSCI.2984-16.2016 PMC5299565

[24] Krishnan GP, Rosen BQ, Chen J-Y, Muller L, Sejnowski TJ, Cash SS, Halgren E, Bazhenov M, Thalamocortical and intracortical laminar connectivity determines sleep spindle properties, PLoS Comput. Biol 14 (6) (2018) e1006171. 29949575 10.1371/journal.pcbi.1006171 PMC6039052

[25] Wei Y, Krishnan GP, Bazhenov M, Synaptic mechanisms of memory consolidation during sleep slow oscillations, J. Neurosci 36 (15) (2016) 4231–4247. 27076422 10.1523/JNEUROSCI.3648-15.2016 PMC4829648

[26] Wei Y, Krishnan GP, Komarov M, Bazhenov M, Differential roles of sleep spindles and sleep slow oscillations in memory consolidation, PLoS Comput. Biol 14 (7) (2018) e1006322. 29985966 10.1371/journal.pcbi.1006322 PMC6053241

[27] Rosen BQ, Krishnan G, Sanda P, Komarov M, Sejnowski T, Rulkov N, Ulbert I, Eross L, Madsen J, Devinsky O, , Simulating human sleep spindle MEG and EEG from ion channel and circuit level dynamics, J. Neurosci. Methods 316 (2019) 46–57. 30300700 10.1016/j.jneumeth.2018.10.002 PMC6380919

[28] Wei Y, Krishnan GP, Marshall L, Martinetz T, Bazhenov M, Stimulation augments spike sequence replay and memory consolidation during slow-wave sleep, J. Neurosci 40 (4) (2020) 811–824. 31792151 10.1523/JNEUROSCI.1427-19.2019 PMC6975295

[29] González OC, Sokolov Y, Krishnan GP, Delanois JE, Bazhenov M, Can sleep protect memories from catastrophic forgetting? ELife 9 (2020) e51005. 32748786 10.7554/eLife.51005 PMC7440920

[30] Sanda P, Malerba P, Jiang X, Krishnan GP, Gonzalez-Martinez J, Halgren E, Bazhenov M, Bidirectional interaction of hippocampal ripples and cortical slow waves leads to coordinated spiking activity during nrem sleep, Cerebral Cortex 31 (1) (2021) 324–340. 32995860 10.1093/cercor/bhaa228 PMC8179633

[31] Hines M, Davison AP, Muller E, Neuron and python, Front. Neuroinform 3 (2009) 391. 10.3389/neuro.11.001.2009 PMC2636686 19198661

[32] Lytton WW, Seidenstein AH, Dura-Bernal S, McDougal RA, Schürmann F, Hines ML, Simulation neurotechnologies for advancing brain research: parallelizing large networks in neuron, Neural Comput. 28 (10) (2016) 2063–2090. 27557104 10.1162/NECO_a_00876 PMC5295685

[33] Sivagnanam S, Majumdar A, Yoshimoto K, Astakhov V, Bandrowski AE, Martone ME, Carnevale NT, , Introducing the neuroscience gateway, IWSG 993 (2013). 10.1002/cpe.3283 PMC4624199 26523124

[34] Marru S, Pierce M, Plale B, Pamidighantam S, Wannipurage D, Christie M, Ranawaka I, Abeysinghe E, Quick R, Tajkhorshid E, , Cybershuttle: An end-to-end cyberinfrastructure continuum to accelerate discovery in science and engineering, in: Practice and Experience in Advanced Research Computing, 2023, pp. 26–34.

[35] Lindén H, Hagen E, Leski S, Norheim ES, Pettersen KH, Einevoll GT, LFPy: a tool for biophysical simulation of extracellular potentials generated by detailed model neurons, Front. Neuroinform 7 (2014) 41. 24474916 10.3389/fninf.2013.00041 PMC3893572

[36] Staresina BP, Coupled sleep rhythms for memory consolidation, Trends in Cognitive Sciences (2024). 10.1016/j.tics.2024.02.002 38443198

[37] Petzka M, Chatburn A, Charest I, Balanos GM, Staresina BP, Sleep spindles track cortical learning patterns for memory consolidation, Curr. Biol 32 (11) (2022) 2349–2356. 35561681 10.1016/j.cub.2022.04.045 PMC9616732

[38] Yordanova J, Kirov R, Verleger R, Kolev V, Dynamic coupling between slow waves and sleep spindles during slow wave sleep in humans is modulated by functional pre-sleep activation, Sci. Rep 7 (1) (2017) 14496. 29101344 10.1038/s41598-017-15195-x PMC5670140

[39] Golden R, Delanois JE, Sanda P, Bazhenov M, Sleep prevents catastrophic forgetting in spiking neural networks by forming a joint synaptic weight representation, PLoS Comput. Biol 18 (11) (2022) e1010628. 36399437 10.1371/journal.pcbi.1010628 PMC9674146

[40] Durán E, Pandinelli M, Logothetis NK, Eschenko O, Altered norepinephrine transmission after spatial learning impairs sleep-mediated memory consolidation in rats, Sci. Rep 13 (1) (2023) 4231. 36918712 10.1038/s41598-023-31308-1 PMC10014950

[41] de Quervain D, Schwabe L, Roozendaal B, Stress, glucocorticoids and memory: implications for treating fear-related disorders, Nat. Rev. Neurosci 18 (1) (2017) 7–19. 27881856 10.1038/nrn.2016.155

[42] Power JM, Sah P, Nuclear calcium signaling evoked by cholinergic stimulation in hippocampal CA1 pyramidal neurons, J. Neurosci 22 (9) (2002) 3454–3462. 11978822 10.1523/JNEUROSCI.22-09-03454.2002 PMC6758367

[43] Hasselmo ME, Alexander AS, Hoyland A, Robinson JC, Bezaire MJ, Chapman GW, Saudargiene A, Carstensen LC, Dannenberg H, The unexplored territory of neural models: Potential guides for exploring the function of metabotropic neuromodulation, Neuroscience 456 (2021) 143–158. 32278058 10.1016/j.neuroscience.2020.03.048 PMC7541517

[44] Kumbhar P, Hines M, Fouriaux J, Ovcharenko A, King J, Delalondre F, Schürmann F, Coreneuron: An optimized compute engine for the neuron simulator, Front. Neuroinform 13 (2019) 63. 31616273 10.3389/fninf.2019.00063 PMC6763692

[45] Mainen ZF, Sejnowski TJ, Influence of dendritic structure on firing pattern in model neocortical neurons, Nature 382 (6589) (1996) 363–366. 8684467 10.1038/382363a0

